# Development of a theoretical model for upright postural control in lower limb prosthesis users

**DOI:** 10.1038/s41598-021-87657-2

**Published:** 2021-04-15

**Authors:** David F. Rusaw, Rasmus Alinder, Sigurd Edholm, Karin L. L. Hallstedt, Jessika Runesson, Cleveland T. Barnett

**Affiliations:** 1 School of Health and Welfare, Jönköping University, Box 1026, 55111 Jönköping, Sweden; 2 School of Science and Technology, Nottingham Trent University, Nottingham, UK

**Keywords:** Biomedical engineering, Health care

## Abstract

Methods used to assess quiet standing in unilateral prosthesis users often assume validity of an inverted pendulum model despite this being shown as invalid in some instances. The aim of the current study was to evaluate the validity of a proposed unilaterally-constrained pin-controller model in explaining postural control in unilateral prosthesis users. Prosthesis users were contrasted against the theoretical model as were able-bodied controls that stood on a platform which unilaterally constrained movement of the CoP. All participants completed bouts of quiet standing with eyes open, eyes closed and with feedback on inter-limb weight bearing asymmetry. Correlation coefficients were used to infer inverted pendulum behavior in both the anteroposterior and mediolateral directions and were derived from both kinematic (body attached markers) and kinetic (centre of pressure) experimental data. Larger, negative correlation coefficients reflected better model adherence, whilst low or no correlation reflected poorer model adherence. Inverted pendulum behavior derived from kinematic data, indicated coefficients of high magnitude in both mediolateral (all cases range 0.71–0.78) and anteroposterior (0.88–0.91) directions, irrespective of groups. Inverted pendulum behavior derived from kinetic data in the anteroposterior direction indicated validity of the model with large negative coefficients associated with the unconstrained/intact limbs (prosthesis users: − 0.45 to − 0.65, control group: − 0.43 to − 0.72), small coefficients in constrained/prosthetic limbs (prosthesis users: − 0.02 to 0.07, control group: 0.13–0.26) and large negative coefficients in combined conditions (prosthesis users: − 0.36 to − 0.56, control group: − 0.71 to − 0.82). For the mediolateral direction, coefficients were negligible for individual limbs (0.03–0.17) and moderate to large negative correlations, irrespective of group (− 0.31 to − 0.73). Data suggested both prosthesis users’ and able-bodied individuals’ postural control conforms well to that predicted by a unilaterally-constrained pin-controller model, which has implications for the fundamental control of posture in transtibial prosthesis users.

## Introduction

A link between fear of falling and postural control has been established in young able-bodied individuals^1^ and self-reported measures of falls efficacy have been shown to be linked to prospective fall risk in older individuals^2^. Following amputation surgery, prosthesis users face problems with balance and postural control^3^ and over half of prosthesis users report to have fallen in a preceding 1-year period^4,5^. Their increased fear of falling can lead to reduced social participation^6^. Although not yet mechanistically understood, postural control has recently been shown to be linked to and predictive of falls efficacy in transtibial prosthesis users^5^. This may partially explain the increased risk and fear of falling in this group^4^, although these postural control measures are relatively static in nature and falls may also occur during dynamic activities^5^. Nonetheless, research that can explain and improve the understanding of the underlying mechanisms of balance and postural control in prosthesis users, would significantly improve their health and well-being. However, methods used to investigate prosthesis users’ balance and postural control should account for the often asymmetrical nature of their function.

Both dynamic and more static measures of balance and postural have been used to characterize the performance of prosthesis users^7^. However, such measures often have implicit assumptions associated with the inverted pendulum model of upright posture. That is to say, they do not account for the often asymmetrical nature prosthesis users’ balance function. The inverted pendulum model predicts the relationship between the centre of pressure (CoP) and centre of mass (CoM) to be a negative correlation between the difference in CoP-CoM position and the horizontal CoM acceleration in the same plane in both the anteroposterior and mediolateral directions^8–10^. This model provides a useful theoretical framework with which to explore human postural control. The inverted pendulum model has been explored extensively and modifications to it have been proposed, for example utilizing more than a single body segment^11–14^. Despite these efforts, methods employing a single segment inverted pendulum model during quiet standing are still utilized and have been shown, both historically^8–10^, and more recently^15,16^, to be valid for able-bodied individuals. When applied to individuals with unilateral limb loss, a key of limitation of the inverted pendulum models is the lack of accounting for the different behaviour of the biological limbs and prosthetic components. Previous research has confirmed that for prosthesis users, there is a clear inter-limb asymmetry in mechanical function that may render the direct application of this single inverted pendulum model to this patient group problematic^17,18^. For this reason, it is necessary to establish new models of postural control that reflect this unilateral difference in function. These models must also be robust to sensory perturbations, given that unilateral prosthesis users have been shown to heavily rely on visual information^7,19^ and display inter-limb weight-bearing^7,17,19,20^ during balance tasks.

Previous research has shown that unilateral transtibial prosthesis users (TPU) rely more on the intact limb than the affected prosthetic limb for control of upright posture^7,20–22^. This suggests that each limb plays a unique role in unilateral TPU participants. The intact limb seems to perform the role of a ‘controller’, utilising increased strength and range of motion to perform corrective postural movements^7,20–22^. The affected limb and prosthetic componentry, however, act as a relatively rigid ‘pin’ with negligible COP movement, potentially due to lack of control^7,20–22^. Recent efforts to explore if unilateral prosthesis users can utilize unilateral control in inverted pendulum-based postural control have revealed that they have the ability via residual musculature on the amputated side^23,24^ and the passive qualities of the prosthetic componentry^21,25^. Although ankle stiffness has been shown to affect postural control in able-bodied individuals^26,27^, the extent of passive stiffness of the prosthetic foot on postural control of prosthesis users remains largely unknown. Developing a new model that accounts for the unilateral constraints observed in prosthesis users’ postural control and is robust to sensory perturbations would provide a theoretical and experimental framework for this future research. By assessing each limb separately, such models would be useful when investigating the effects of prosthetic component design, particularly in devices with control functions aimed at improving postural control. Similarly, inter-limb differences in function arising during or following physical therapy that attempts to improve balance would be better understood.

Therefore, the current study aimed to develop and assess the efficacy of a theoretical unilaterally-constrained model to represent control of upright posture in unilateral TPUs. The study had two specific objectives: (1) To empirically establish how closely the predicted outcomes of upright postural control from a unilaterally-constrained model matched the measured outcomes from a group of unilateral TPUs and a group able-bodied individuals with mechanical constraints on the CoP; and, (2) to assess how closely matched the measured outcomes of upright postural control were between a group of unilateral TPUs and a group able-bodied individuals with mechanical constraints on the CoP.

It was hypothesised that: (1) magnitude and direction of correlation coefficients from unilateral TPUs’ upright postural control, would closely match those predicted by a unilaterally constrained model; (2) magnitude and direction of correlation coefficients from able-bodied participants upright postural control, with constraints on CoP movement, would closely match those predicted by a unilaterally constrained model; (3) magnitude and direction of correlation coefficients from able-bodied participants upright postural control, under CoP constraint, would match those from the unilateral TPU group; and, (4) that the effects described above (hypotheses 1–3), would be robust in response to sensory perturbations and provision of visual feedback.

## Methods

### Participants

Two groups were recruited for the current study using a consecutive sampling method. A convenience sample of unilateral TPUs (n = 8) was recruited from local prosthetic clinics (Table 1). Participants in the TPU group were included if they were able to; use their prosthesis without pain or discomfort, had used a prosthesis for over one year prior to testing, and were able to remain standing for periods of at least one minute at a time to complete the experimental protocol. TPU participants were excluded if; they had current concomitant health issues, ongoing issues with the contralateral and/or residual limb or were taking medication known to affect balance and postural control. An able-bodied group (AB, n = 7) matched for height, mass, age and gender were also recruited (Table 1). The study was carried out in accordance with all relevant local guidelines and regulations approval was granted by the regional ethical review board in Linköping, Sweden (2013/135-31) and all participants gave written informed consent to participate in the study.

**Table 1:** Participant characteristics for the two groups (TPU: left; AB: right). Descriptive data for: sex, height (cm), weight (kg), age (years), time since amputation (years), residual limb length (cm), proximal residual limb circumference (cm), distal residual limb circumference (cm), cause of amputation, residual limb length classification (modified from: ^28^), foot (foot classification^29^), suspension (vacuum—elevated vacuum; pin—pin suspension), Liner (TPE—thermoplastic elastomer, silicon—silicon liner), socket (TC—total contact; PTB—patellar tendon-bearing). Numerical data presented with mean and standard deviation (SD).

TPU-participant	Sex	Height (cm)	Weight (kg)	Age (years)	Time since amputation (years)	Residual limb length (cm)	Proximal limb circumference (cm)	Distal limb circumference (cm)	Cause of amputation	Residual limb length classification	Foot	Suspension	Liner	Socket	AB-participant	Sex	Height (cm)	Weight (kg)	Age (years)
1	M	182	82	68	35	18	36	28	Trauma	Ordinary	ESAR	Vacuum	TPE	TC	1	M	183.5	80	53
2	M	174	72	72	18	18	31	26	Trauma	Ordinary	ESAR	Pin	TPE	PTB	2	M	177.5	83	39
3	M	171	113	73	25	19	36	28	Trauma	Ordinary	ESAR	Vacuum	TPE	TC	3	M	180	91.5	73
4	M	182	54	63	23	17	30	24	Vascular	Ordinary	ESAR	Pin	TPE	PTB	4	M	190	74	23
5	M	182	81.5	37	17	14	35	29	Trauma	Ordinary	ESAR	Vacuum	TPE	TC	5	M	174	81	71
6	M	183	75	51	7	19	32	27	Trauma	Ordinary	ESAR	Vacuum	TPE	TC	6	M	176	66	63
7	M	191	82	22	6	19	38	28	Sarcoma	Ordinary	ESAR	Vacuum	TPE	TC	7	M	178.5	92	45
8	M	180	90	46	28	22	35	23	Trauma	Ordinary	ESAR	Vacuum	Silicon	TC	8	M			
Mean (SD)		180.6 (5.7)	81.1 (15.6)	54.0 (17.1)	19.9 (10.0)	18.3 (2.3)	34.1 (2.8)	26.6 (2.1)									179.9 (5.0)	81.1 (8.5)	52.4 (16.8)

### Unilaterally-constrained pin-controller model

**Figure 1:**
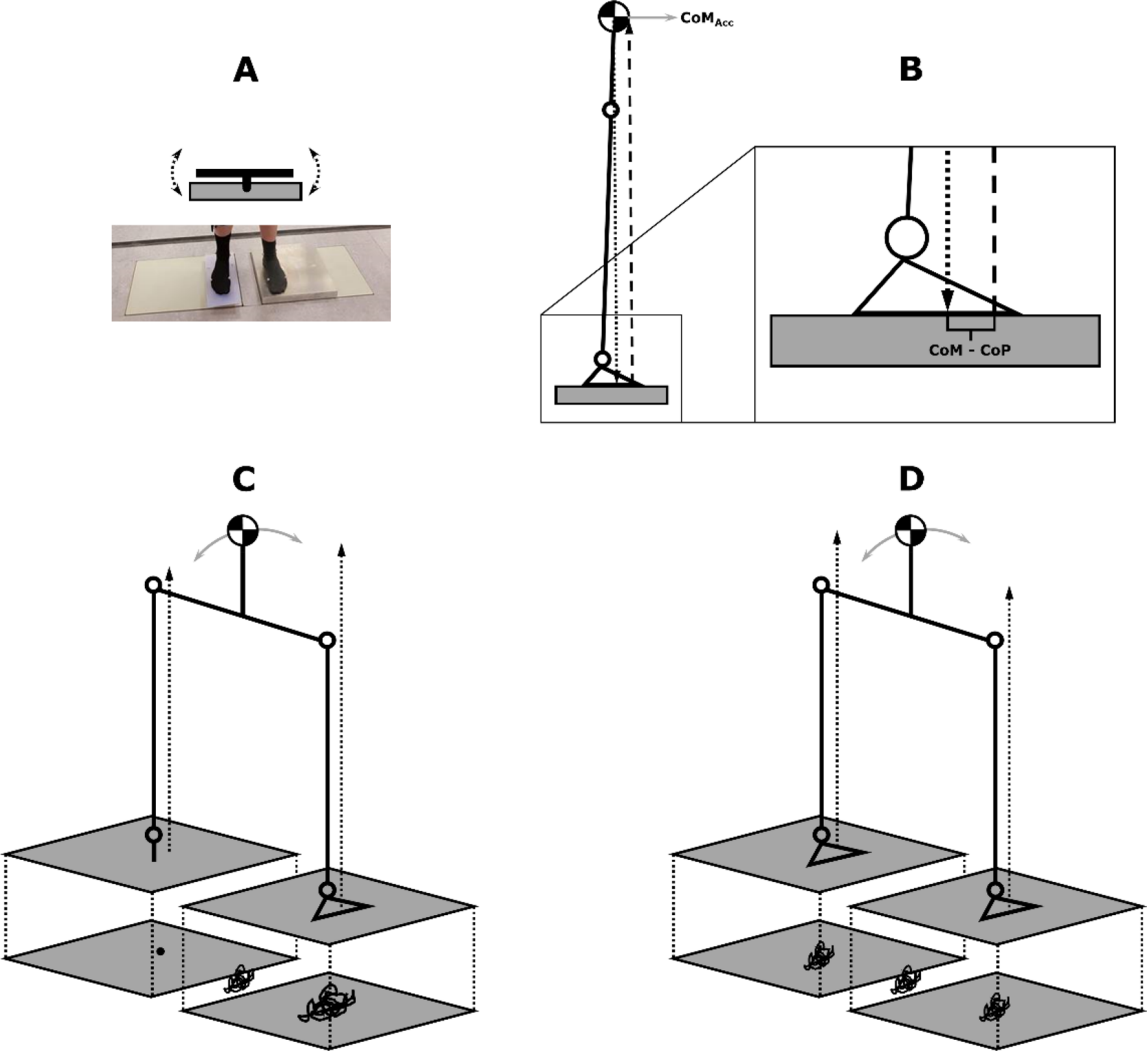
Schematic illustrations of the theoretical models and apparatus used in the current study. The device used to constrain the control participants’ CoP under the right foot (**A**). A solid aluminium plate with a pinhead underneath forming a ball-and-socket articulation with an indentation in a separate solid block that was rigidly attached to the force platform below. The inverted pendulum model (**B**) with inputs of centre of mass (CoM) and centre of pressure (CoP) positions and the centre of mass acceleration (CoM_Acc_). Illustration of bipedal stance of a unilateral lower limb prosthesis user (**C**) and control (**D**) with CoP data recorded from each foot placed on force plates arranged in parallel. The model predicts that in scenario C, the affected limb (left side) acts a pin with the intact limb the controller, with lower and higher correlations of CoM-CoP with the CoM_Acc_, respectively. Height adjustments made in offline analysis to account for the control groups’ experimental apparatus.

The proposed theoretical unilaterally-constrained pin-controller model is based upon kinetic observations that the control of the CoP and CoM from the affected limb is reduced and potentially negligible when compared to the intact limb. For example, the CoP under the intact limb is more likely to be highly negatively correlated with whole-body CoM acceleration in both anteroposterior and mediolateral directions, as is observed in the conventional inverted pendulum model of human upright posture^8^. This negative correlation would suggest that the intact limb provides most of the control of whole-body CoM. Conversely, where the affected limb does not have a significant role in the control of whole-body CoM kinematics, then the CoP under the intact will result in a low or no correlation (Fig. 1).

### Physical constraint on postural control

To assess the assumptions and further validate the theoretical model, independent of lower limb amputation, a device that physically constrained the CoP position was constructed (Fig. 1A). This device comprised a three degree of freedom pin articulation located underneath a solid footplate. A pinhead on the footplate formed a ball and socket articulation with a convex indentation within a solid floor block which was rigidly affixed to a force platform (Fig. 1). Compensations made for height differences between the force platforms and the experimental manipulation for the control group were as follows: as the overall height of the aluminium block and the footplate were the same, prior to data collection, four additional reflective markers were used to note the planar location of the aluminium block. Following data collection, a series of ‘force structures’, were created to account for the height differences. The constrained limbs CoP was extracted from the height of the forceplate. The unconstrained limbs CoP was extracted from the force structure constructed with the use of the four markers placed earlier. The remaining force structure with the combined CoP was derived from the resultant CoP from the unconstrained CoP (aluminium block with force structure) and constrained CoP (force platform projected onto combined force structure) together. Previous research has reported that the CoP is shifted anteriorly under the prosthetic foot^18,30^. Therefore, when positioning the able-bodied participants’ right foot on the footplate, the axis of the pin joint was aligned to 66% of the distance from the ankle joint centre (lateral malleoli) to the distal head of the 5th metatarsal in the sagittal plane. The contralateral foot was positioned according to the previously noted stance width. Able-bodied participants were required to complete trials using this device, avoiding footplate contact with the ground, which was verified by assessing CoP position deviation from zero.

### Experimental design

Participants completed trials comprising of 60 s of upright postural control i.e. quiet standing, in three conditions. Trials with normal vision (eyes open—EO), were conducted to assess the model efficacy under typical visual conditions. Trials were also conducted under perturbed visual conditions (eyes closed—EC) to assess model efficacy independent of visual input, as TPUs have been shown to rely heavily on vision during postural control^7,19^. Finally, to assess model efficacy independent of inter-limb weight-bearing asymmetry, often observed in TPUs^18,20^, trials were recorded as participants received feedback regarding weight-bearing (weight-bearing feedback—FB). In the FB condition, participants were instructed to maintain as close to a 50:50 weight distribution as possible, as a custom program displayed real-time inter-limb vertical ground reaction force distribution from the two parallel force plates (Labview (v12.0, National Instruments Corporation, Inc.; Austin, USA)^18^. Ordering of the EO and EC trials were randomised, with the FB condition always last.

### Experimental protocol

At the start of each data collection session, the position of the force plates and subsequent CoP locations were located and verified in the global laboratory coordinate system using a previously reported procedure, ensuring spatial synchronisation^31^. Prior to trial commencement, foot position and mediolateral base of support were determined by asking participants to march on the spot for 10 s, stop and then take up a comfortable posture, at which point the distance between the medial malleoli was noted and maintained during all trial data collection. Participants were fitted with a safety harness, and positioned in the capture volume, standing with each foot on one of two force plates (model BP400600, AMTI, Inc.; Watertown, USA). Participants were then asked to stand comfortably and remain still, facing forwards towards a screen 3 m away which was blank for EO and EC conditions and displaying weight-bearing information for the FB condition. Participants maintained this position for 60 s, at which point they were free to move and rest, before being re-positioned for the next trial. Three trials for each of the three conditions were completed (n = 9 trials in total)^18,32^. Participants received no practice attempts and were afforded rest periods, ad libitum. A 13-segment whole-body model (head, upper and lower arms, thorax, pelvis, thigh, shank, and feet) was defined using 69 passive-reflective markers^17,32^. Kinematics were collected using an 11-camera motion capture system (model Oqus; Qualisys AB; Gothenburg, Sweden). All data were sampled at 100 Hz and all raw data exported in .c3d format for post-processing.

### Data analysis

Centre of pressure data were recorded from two force plates resulting in CoP under each limb for both groups. A global CoP was derived using combined CoP signals of both force plates. Whole-body CoM location was computed using the full-body kinematic data and anthropometric data^33^ in Visual 3D (C-Motion, Germantown, US). These data were then used to define both kinetic (CoP) and kinematic (body markers) models to allow computation of the outcome variables described below, using the full 60-s trial length. Firstly, correlation coefficient [Pearson’s Product Moment (r)] between CoM–CoP distance under each foot/combined CoP and CoM acceleration (CoM_Acc_) in the mediolateral and anteroposterior directions were calculated to indicate model efficacy of the unilaterally-constrained pin-controller model (Fig. 1). The proposed unilaterally-constrained pin-controller model predicts that analysis of the intact limb (unconstrained side in the control group) would result in a high negative correlation i.e. coefficient values of − 1 and on the prosthetic side (constrained side in the control group) low or no correlation i.e. coefficient values near 0 (Fig. 1). In addition, the same correlation coefficient was calculated for the mean height above support surface for each marker and their total linear movement in mediolateral and anteroposterior directions, as per the kinematic definition. It has been shown that prosthesis users have similar kinematic but different kinetic inverted pendulum behaviour in quiet standing when compared to able-bodied individuals^18^. Therefore, analysis of kinematic inverted pendulum behaviour was conducted to ensure that this current validation reflected kinetic inverted pendulum behaviour and not a new, unforeseen kinematic consequence of the constraining intervention.

### Statistical analysis

Independent variables of Group (between-subject factors: TPU or AB), sensory conditions (within-subject factors: EO, EC, FB) and CoP under each limb (Limb) (within-subject factors: constrained/prosthesis, unconstrained/intact, total) were defined. Unless otherwise stated, statistical comparisons were prosthesis vs. constrained and intact vs. unconstrained^18^. Normality of data were assessed using a Shapiro–Wilk test. A Greenhouse–Geisser adjustment was used where violations of the assumption of sphericity were present. Multiple posthoc comparisons were adjusted using a Bonferroni correction. Descriptive statistics (mean and SD) and 95% confidence intervals were calculated for all correlation coefficients. To address hypotheses 1, 2 and 3, one three-way mixed ANOVA was used to compare mean coefficient for CoM-CoP distance under each foot/combined CoP and the CoM acceleration (CoM_Acc_) in the mediolateral and anteroposterior directions; and, to validate the kinematic inverted pendulum model as explained in the data analysis section, one two-way mixed ANOVA was used to compare mean coefficient for height above support surface for each marker and their total linear movement in mediolateral and anteroposterior directions.

## Results

All correlation coefficients [Pearson’s Product Moment (r)], are presented in Table 2. These indicate the strength of relationships between the proposed models and observed experimental behaviour.

**Table 2:** Mean and 95% confidence intervals of the back-transformed correlation coefficients of the direction (AP = anteroposterior, ML = mediolateral), group (transtibial prosthesis users = TPU or able-bodied = AB), sensory conditions (eyes-open = EO, eyes-closed = EC, feedback = FB) and CoP under each limb (Limb) (PROS = constrained/prosthesis, INTACT = unconstrained/intact, TOTAL).

Kinetics			TPU		AB	
Direction	Limb	Condition	Mean	95% CI	Mean	95% CI
AP	INTACT	EC	− 0.65	− 0.84 to − 0.22	− 0.72	− *0.88* to − *0.33*
		EO	− 0.47	− 0.79 to 0.36	− 0.60	− 0.82 to − 0.09
		FB	− 0.45	− 0.80 to 0.47	− 0.43	− 0.62 to − 0.16
	PROS	EC	− 0.02	− 0.26 to 0.29	0.26	0.18 to 0.35
		EO	0.05	− 0.33 to 0.64	0.22	0.11 to 0.36
		FB	0.07	− 0.27 to 0.57	0.13	0.04 to 0.24
	TOTAL	EC	− 0.56	− 0.81 to 0.03	− 0.82	− 0.93 to − 0.48
		EO	− 0.36	− 0.69 to 0.34	− 0.72	− 0.91 to − 0.14
		FB	− 0.54	− 0.84 to 0.36	− 0.71	− 0.86 to − 0.37
ML	INTACT	EC	0.03	− 0.09 to 0.16	0.07	− 0.03 to 0.18
		EO	0.06	− 0.08 to 0.22	0.06	0.02 to 0.10
		FB	0.08	− 0.11 to 0.32	0.11	0.00 to 0.23
	PROS	EC	0.15	0.04 to 0.28	0.13	0.04 to 0.23
		EO	0.16	− 0.08 to 0.47	0.10	0.04 to 0.16
		FB	0.17	0.00 to 0.36	0.20	0.12 to 0.29
	TOTAL	EC	− 0.73	− 0.93 to 0.01	− 0.45	− 0.72 to 0.08
		EO	− 0.58	− 0.83 to 0.05	− 0.31	− 0.62 to 0.26
		FB	− 0.63	− 0.87 to 0.06	− 0.43	− 0.62 to − 0.15

Kinematics		TPU		AB		
Direction	Condition	Mean	95% CI	Mean	95% CI	
AP	EC	0.89	0.85 to 0.93	0.90	0.87 to 0.93	
	EO	0.91	0.89 to 0.93	0.90	0.85 to 0.95	
	FB	0.88	0.82 to 0.94	0.88	0.80 to 0.96	
ML	EC	0.73	0.70 to 0.77	0.78	0.70 to 0.86	
	EO	0.72	0.67 to 0.76	0.75	0.67 to 0.82	
	FB	0.70	0.65 to 0.76	0.77	0.69 to 0.84	

### Kinetic model definition: mediolateral direction

Correlations derived from the kinetic data were found to be non-normal and a log transformation was conducted on this data set for analysis. There was no statistically significant three-way interaction between Group-Condition-Limb, *F* (2.016, 26.212) = 1.216, *p* = 0.313, partial η^2^ = 0.086, ε = 0.504. There were no statistically significant two-way interactions between Group-Condition or Group-Limb. There was a statistically significant two-way interaction between Condition-Limb, *F*(2.016, 26.212) = 6.096, *p* = 0.007, owing to the larger negative correlation coefficients associated with the ‘total’ vs. other limb factors (Table 2).

### Kinetic model definition: anteroposterior direction

There was no statistically significant three-way interaction between Group-Condition-Limb, *F* (2.374, 30.858) = 2,511, *p* = 0.089, partial η^2^ = 0.086, ε = 0.593. There was no statistically significant two-way interaction between Group-Condition. There was a statistically significant two-way Group-Limb interaction effect, *F*(1.735, 30.858) = 9.400, *p* = 0.002 (Table 2). This arose from the negligible to more positive correlation coefficients associated with the prosthetic/constrained limb factor vs. intact/unconstrained and total limb factors, particularly in the AB group. This observation also resulted in a statistically significant two-way Condition-Limb interaction effect, *F*(2.374, 30.858) = 8.793, *p* = 0.001 (Table 2).

### Kinematic model definition: mediolateral direction

There was no statistically significant interaction between the Group and Condition on coefficients in the ML direction, *F*(2, 26) = 0.537, *p* = 0.591, partial η^2^ = 0.040. There were no statistically significant main effect of Group or Condition.

### Kinematics: anteroposterior direction

There was no statistically significant interaction between the Group and Condition on coefficients in the AP direction, *F*(2, 24) = 0.671, *p* = 0.466, partial η^2^ = 0.053. There were no statistically significant main effect of Group or Condition.

## Discussion

The current study attempted to develop and assess the efficacy of a theoretical unilaterally-constrained pin-controller model, to represent upright postural control in unilateral TPUs. Predicted postural control outcomes from a unilaterally-constrained model closely matched the observed outcomes in both TPUs and an AB group with mechanical constraints on the CoP. Similarly, observed outcomes were similar in TPUs when compared to an AB group with mechanical constraints on the CoP, with some exceptions discussed below.

The hypotheses that observed measures of upright postural control from both unilateral TPUs (1) and an AB group with constraints on CoP movement (2) would closely match those predicted by the unilaterally-constrained model were largely supported. Results reported in the current study seem to support that correlation coefficients associated with the intact/unconstrained limbs were larger and more negative when compared to coefficients from the prosthetic/constrained limbs, which were around zero and, in some cases slightly positive. This suggests that both groups were represented well by the proposed model. The hypotheses (1 and 2) appear to be supported statistically by the lack of three-way interaction effects and a general absence of two-way interaction effects. Collectively, this suggests the proposed model has validity and explains postural control in these groups well. Given the small sample size of this exploratory study, the statistical analysis of correlation coefficients and the resulting large confidence intervals, the authors do wish to caution the reader to draw definitive conclusions from the current study.

**Figure 2:**
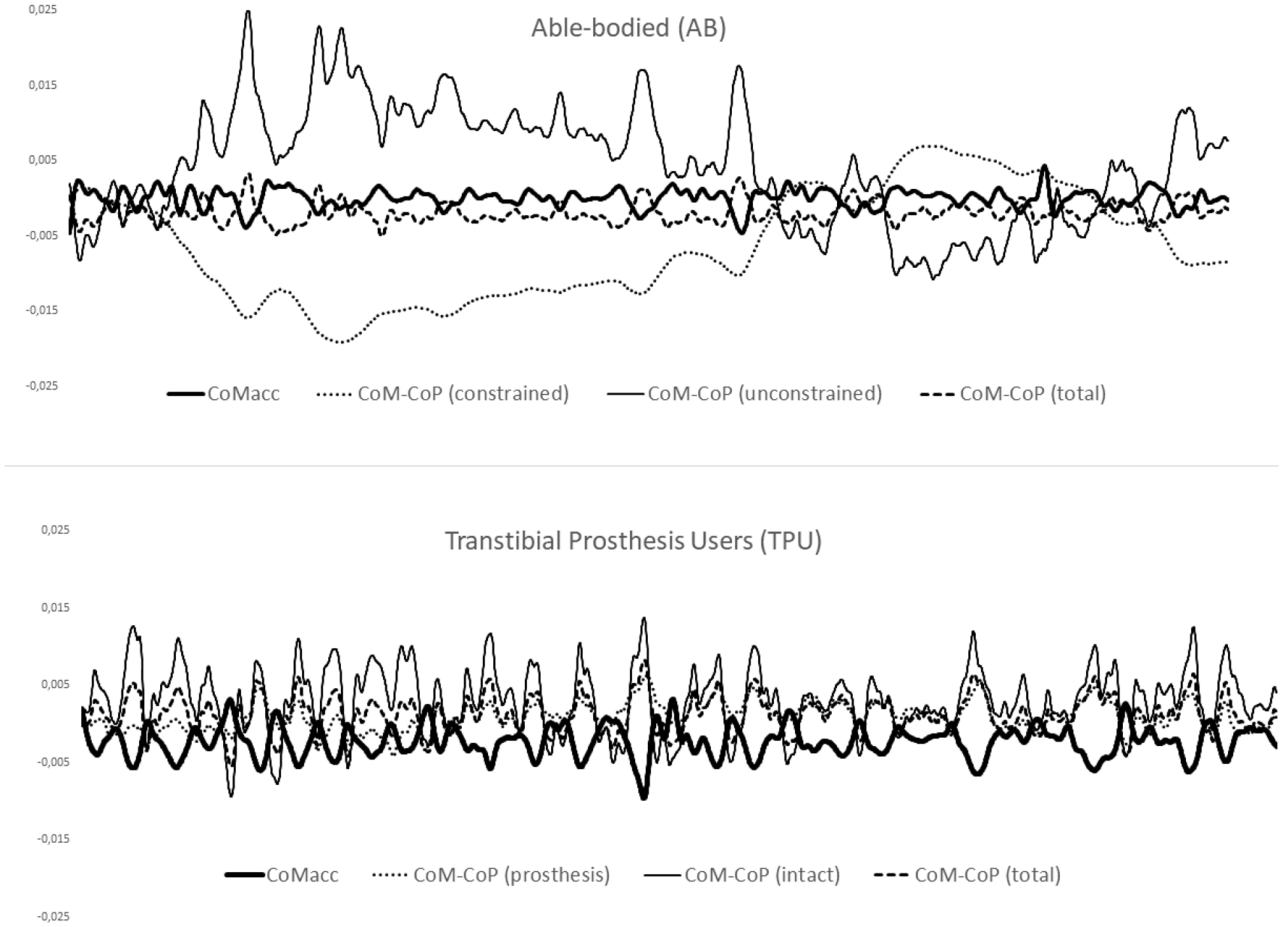
30-s representative time curve for the sagittal plane (anteroposterior) for the able-bodied group (AB) and the transtibial prosthesis user group (TPU). Outcomes in the curve are centre of mass acceleration (CoM_Acc_), difference between the position of the centre of mass and centre of pressure (CoM-CoP) for each of the three sides of interest (prosthetic limb/constrained for AB group), intact limb/unconstrained for AB group, and total). Given the coordinate system utilized (positive = anterior), negative values indicate the CoM is posterior to the CoP.

There were some challenges to the validity of the model. The presence of a significant two-way condition by limb interaction in the anteroposterior direction is at odds with hypotheses 1–3. This may be due to a disproportionate influence of the limb effect. Weaker and more positive correlation coefficients (0–0.2) were observed in the prosthetic/constrained limbs when compared to both the intact/unconstrained and combined ‘total’ limbs. This was particularly evident in the AB group. This positive correlation coefficient in the AB group warrants further exploration. One explanation may be that this is the result of CoM dynamics which are driven primarily by the intact limb. As the intact limb CoM–CoP and CoM_Acc_ are highly negatively correlated, whenever the CoM_Acc_ crosses zero, a positive coefficient in the constrained limb will result, as CoP movement was not possible. TPUs could potentially utilize forefoot stiffness and prosthesis deformation to shift the CoP about the neutral point (zero in the time curve) whereas the AB group had no such possibility. Further, dampening from the prosthetic foot would have affected both the TPUs CoM progression in the anteroposterior direction and the movement of the CoP in the TPU group. Although the movement of the CoP under the prosthetic foot is known to be very small^19,30,34^ this analytical difference between the two groups must be acknowledged. An additional explanation is that as the combined ‘total’ coefficient is dictated in part by the magnitude of the difference between the CoM–CoP, a distance which appears larger in AB than TPU (Fig. 2), the resulting coefficient could still be highly correlated owing in at least part to how correlation coefficients are derived.

Previous research investigating upright postural control has reported a negligible correlation in the mediolateral direction for individual limbs^9^. This was also the case in the current study, with both groups exhibiting near-zero coefficients for each limb. Similarly, when viewing the correlation for combined limbs, the coefficients were larger, negative values, which is also in agreement with earlier literature^9,10,18^. Together, these results indicate that the proposed unilaterally-constrained pin-controller model explains postural control for both groups in the mediolateral direction. This result is novel in that it is the first instance of a model which accounts for unilateral differences, such as amputation and prosthesis use, being validated in quiet standing in this patient group. Previous research which has proposed an absence of control on the prosthetic side in prosthesis users^7,18,20–22^ now has a plausible explanation as to how prosthesis users compensate and maintain standing balance. This research confirms the presence of and explains different, unilateral contributions to postural control for prosthesis users. Though, the extent of these contributions, or if ability to modify them exists, remains unknown. Previous research has shown that factors originating on the prosthetic side can influence postural control, such as residual musculature^23,24^ and passive stiffness of the prosthetic foot^21,25^. Additionally, for able-bodied individuals, the control of the ankle in postural control is well-established^26,27^. Utilizing the new model, in conjunction with properly-controlled study designs, we can now begin to explore the individual contributions of the intact and prosthetic side on postural control in this subject group. This could be applied to the assessment of prosthetic components effects of on upright posture, particularly those devices whose functionality is aimed at stabilizing the lower limb to improve balance^35^. This would shed light not only on the overall performance but also the relative function of each limb and how the individual is responding to the alterations in device set-up.

A significant interaction effect that challenges support for hypotheses 1–3 is the two-way condition by limb interaction effect in the mediolateral direction. Our results suggest this is due to increased negative magnitudes in the total, combined ‘limb’ condition, with a more pronounced effect in TPUs vs. CON. This suggests that, in an overall sense, TPUs behaved more like inverted pendulums in the mediolateral direction than the control group. This does not refute the hypothesis fully as the inter-limb asymmetry of function is to be expected, hence the justification of the study.

Similarly, the hypothesis (3) that upright postural control would be similar between able-bodied participants under CoP constraint and TPUs was largely supported. As discussed earlier, the lack of three-way and two-way interactions, particularly those contrasts with group factors, suggest validity of the model. One challenge to this outright validity is the significant two-way group by limb interaction effect in the anteroposterior direction. As above, this seems due to the disproportionately dominant limb effect, where weaker (more positive coefficient, 0–0.2) in pros/constrained limb vs intact/unconstrained and total, particularly in AB group were observed.

The final hypothesis (4) that the effects predicted above would be robust in response to sensory perturbations and provision of feedback was supported. Both TPU and AB groups’ postural control conformed to that suggested by the unilaterally-constrained pin-controller model, regardless of whether visual information was present (EO) or not (EC). Good conformation to the model was also observed when feedback allowed both groups to maintain inter-limb symmetry regarding weight-bearing (FB). One interpretation of these consistent effects is that whilst TPUs rely heavily on vision and display asymmetry in weight-bearing during postural control^18–20,36^, affecting the magnitude of postural control i.e. sway, the underlying system control dynamics are unaffected. The independent roles of the intact limb ‘controller’ and affected ‘pin’ limbs suggest avenues for improved control should be explored. For example, increasing affected limb hip strength and flexibility, as well as increasing the controlled articulation/deformation of the prosthetic ankle–foot components in TPUs, may restore pseudo hip and ‘ankle’ strategies, allowing for greater control of CoP and CoM displacements.

Finally, the current study demonstrates that aggregating data between limbs in TPUs is inappropriate and further confirms the need for independent assessment of both the affected and intact limbs when investigating postural control in TPUs, given they perform very different roles in terms of control^18^. Therefore, future research should attempt to validate the proposed unilaterally constrained pin-controller model for use as a clinically meaningful tool by investigating the extent to which an individual conforms to this model is related to and predicts their fall risk, falls incidence and/or fear of falling, for example.

There were some limitations to the current study. The TPU sample was small in number were relatively young, male, well-experienced prosthesis users, secondary mainly to trauma. Therefore, these exploratory analyses are likely more valid for similar groups of prosthesis users. It remains to be seen whether the model described would be valid in specific groups of prosthesis users e.g. fallers or those secondary to vascular issues. As previously stated, the statistical analysis of correlation coefficients from a small sample with large confidence intervals suggests further research is required to confirm the effects noted. Also, it seems that the method used to constrain CoP movement in the AB group did not exactly match the TPU group where viscoelastic properties of the prosthetic foot may have had an influence. Therefore, the AB group may have been operating under a slightly different set of mechanical constraints on the CoP.

In conclusion, for the intact and unconstrained limbs, the coupling of CoM acceleration with the kinematics of the CoP was much stronger when contrasted against the prosthetic and constrained limbs. These results suggest that both TPUs and able-bodied individuals’ postural control conforms well to that predicted by a unilaterally-constrained pin-controller model. These effects held independent of visual manipulation and weight-bearing asymmetry, suggesting that the proposed model has implications for the fundamental control of posture in transtibial prosthesis users.
